# Biofilms harbour *Clostridioides difficile*, serving as a reservoir for recurrent infection

**DOI:** 10.1038/s41522-021-00184-w

**Published:** 2021-02-05

**Authors:** Charmaine Normington, Ines B. Moura, Jessica A. Bryant, Duncan J. Ewin, Emma V. Clark, Morgan J. Kettle, Hannah C. Harris, William Spittal, Georgina Davis, Matthew R. Henn, Christopher B. Ford, Mark H. Wilcox, Anthony M. Buckley

**Affiliations:** 1 Healthcare-Associated Infections Group, Leeds Institute of Medical Research, Faculty of Medicine and Health, University of Leeds, Leeds, LS1 9JT UK; 2 Microbiome Sciences, Seres Therapeutics Inc., Cambridge, MA USA

**Keywords:** Biofilms, Pathogens

## Abstract

*C. difficile* infection (CDI) is a worldwide healthcare problem with ~30% of cases failing primary therapy, placing a burden on healthcare systems and increasing patient morbidity. We have little understanding of why these therapies fail. Here, we use a clinically validated in vitro gut model to assess the contribution of biofilms towards recurrent disease and to investigate biofilm microbiota-*C. difficile* interactions. Initial experiments show that *C. difficile* cells became associated with the colonic biofilm microbiota and are not depleted by vancomycin or faecal microbiota transplant therapies. We observe that transferring biofilm encased *C. difficile* cells into a *C. difficile* naïve but CDI susceptible model induces CDI. Members of the biofilm community can impact *C. difficile* biofilm formation by acting either antagonistically or synergistically. We highlight the importance of biofilms as a reservoir for *C. difficile*, which can be a cause for recurrent infections.

## Introduction

*Clostridioides difficile* is the leading cause of infective antibiotic-associated diarrhoea worldwide and a significant cause of morbidity and mortality; the burden of healthcare costs are estimated to be over €3B in Europe and $4.8B in USA^1–3^. Antibiotics deplete the intestinal microbiota which allows the germination of *C. difficile* spores followed by *C. difficile* cell proliferation and toxin production. Toxins A (TcdA) and B (TcdB) are responsible for the clinical manifestations of *C. difficile* infection (CDI)^4,5^. The primary treatment option is antibiotic therapy, with either metronidazole, vancomycin or fidaxomicin; however, antibiotic therapy further exacerbates intestinal dysbiosis and potentiates recurrent infection^6^. Approximately 30% of primary CDI cases recur after antibiotic treatment for primary inflection^7^, after which, patients are at an increased risk of further treatment failures. The risk of a second and third recurrent episode increases to 45% and 64%, respectively, known as a ‘recurrence escalator’^8^. Recurrent CDI is particularly problematic for the patient and the healthcare system, increasing patient morbidity, extending the number of bed days and requiring more therapy, thus increasing the cost of treatment^3^.

The majority of recurrent episodes are attributed to the original strain/ribotype^9^, suggesting that *C. difficile* can evade antibiotic treatment, possibly by occupying a protective niche within the intestine where antibiotic therapy is ineffective. Incorporation of *C. difficile* into intestinal biofilms, a known driver of chronic infection^10^, could function as a protective niche where *C. difficile* cells are protected from the effects of antibiotic therapy. In vitro, *C. difficile* forms aggregates enclosed in an extracellular matrix^11–14^ and can interact with other bacterial species found within the intestine to enhance biofilm formation^11,15^. Biofilm-associated *C. difficile* cells undergo metabolic remodelling compared with planktonic-associated cells and have a different array of cell-surface proteins/organelles compared with luminal cells^16^. Indeed, biofilm structures composed of *C. difficile* cells have been observed adjacent to epithelial cells in in vivo models of CDI^17–20^, where damaged and necrotic microvilli have been observed^21^. These biofilm cells are enclosed in a glycan-rich extracellular matrix that helps protect against antibiotic exposure^22^. However, little is known about this potential reservoir, the contribution towards disease recurrence and how other members of the biofilm community interact with *C. difficile*.

**Fig. 1:**
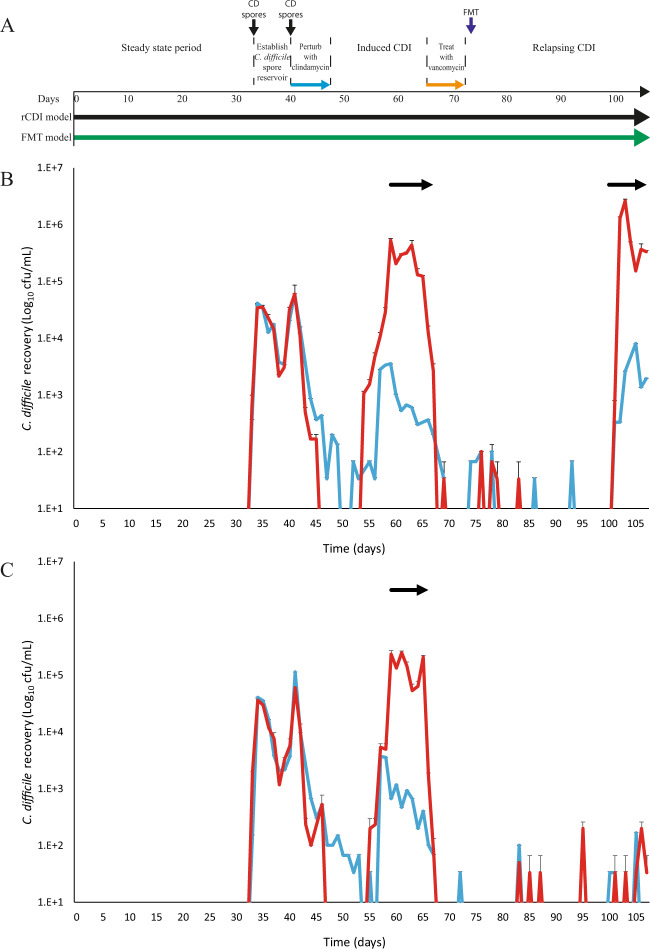
Efficacy of FMT to treat simulated recurrent CDI. **A** Timeline of two in vitro chemostat models that were used to simulate primary CDI and recurrence after vancomycin treatment (black) and vancomycin treatment followed by FMT instillation (green). Luminal *C. difficile* recoveries from the recurrence model (**B**) and from FMT model (**C**). Both figures show the total viable counts (red lines), spores (blue lines) and period of toxin detection (black arrows). Results are shown as mean log_10_ cfu/mL from two biological replicates, and three technical replicates from each. Error bars represent the standard deviation.

We have previously developed a successful in vitro triple-stage chemostat human gut model to evaluate the impact of antimicrobials on intestinal microbiome colonisation resistance to CDI^23^ (Supplementary Fig. 1A). Pooled human faeces are used to establish microbial populations within the gut model. *C. difficile* spores are then added but remain quiescent until the microbial populations and associated colonisation resistance is disrupted, i.e. following antibiotic instillation, which leads to *C. difficile* germination, outgrowth and toxin production (Fig. 1A). Data generated from in vitro gut models have been shown to be clinically reflective with respect to CDI. For example, antibiotics with a high propensity to induce CDI in patients also induce simulated CDI within the gut model^24–26^. Conversely, antibiotics with a lower in vitro propensity to induce simulated CDI are now recognised to have lower CDI risk^23,27^.

Our in vitro model has been fitted with removable biofilm support structures^28^ enabling us to independently delineate the microbiota dynamics of the biofilm and luminal populations. We have previously described and validated the use of our biofilm support structures in our in vitro model (Supplementary Fig. 2)^28^. In this study, we leverage these structures to investigate the role of biofilms in recurrent CDI. Here, we describe the biofilm-associated microbiota dynamics during simulated CDI and recurrent infections, and the interactions between *C. difficile* and members of the colonic biofilm microbiota.

## Results

### Vancomycin therapy and FMT installation are required to prevent recurrent CDI

In two gut models, a CDI recurrence (rCDI) model and a faecal microbiota transplant (FMT) model, we simulated the induction of CDI through the instillation of an induction antibiotic, clindamycin, and administered a ‘treatment’ antibiotic, vancomycin, which is comparable to a clinical setting (Fig. 1). CDI induction was characterised by *C. difficile* spore germination, vegetative cell outgrowth and detection of toxin activity; peak toxin was detected on day 61 at 3.5 log_10_ reciprocal titre in both rCDI and FMT models. Vancomycin successfully reduced the luminal *C. difficile* recoveries to undetectable levels; however, similar to a clinical setting^29^, we detected recurrent CDI in the rCDI model. This was characterised by a second *C. difficile* outgrowth event and the detection of further toxin activity after 28 days (day 100) after vancomycin administration with a peak toxin of 3 log_10_ reciprocal titre (Fig. 1B, red line)^26^.

In the FMT treatment model, we sought to replicate FMT therapy with a 10% w/v faecal slurry instillation from a single healthy donor, simulating the protocol used at the Leeds General Infirmary (U.K.), via the nasal-jejunal route of administration (Supplementary Fig. 1B). FMT therapy is an effective treatment for the resolution of recurrent CDI with a documented success rate of 76.1%^29^. Antibiotic bioassay determination showed an undetectable level of vancomycin in vessel 1 of the gut model at the time of FMT instillation. FMT instillation successfully prevented the recurrence of CDI up to 35 days following cessation of vancomycin (Fig. 1C, blue line). However, *C. difficile* spores were transiently detected post FMT but we did not detect germination or toxin activity.

### *C. difficile* is incorporated into the multispecies biofilm and is not depleted by vancomycin or FMT instillation

**Fig. 2:**
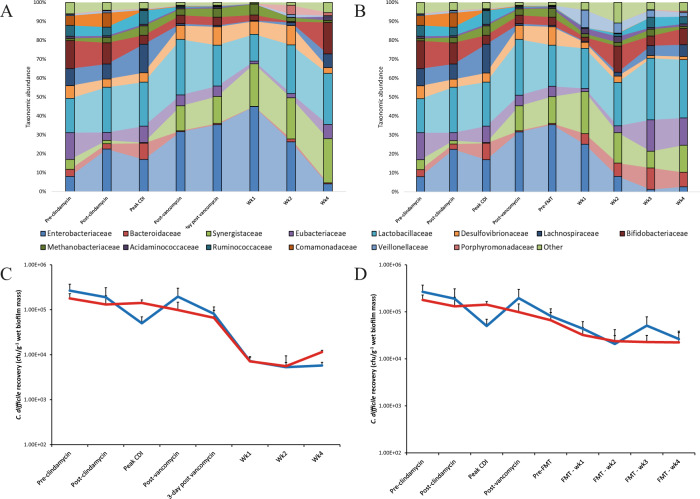
Changes in the biofilm-associated microbiota during CDI and recurrence. Percentage taxonomic abundance of bacterial families isolated from biofilm support structures taken from recurrence (rCDI) model (**A**) or the FMT model (**B**). Graphs constructed using mean (of least 3 support structures/time point) percent abundance of bacterial OTUs assigned to the family taxonomic level. Enumeration of biofilm-associated *C. difficile* (vegetative cells – red lines, spores – blue lines) from support structures from the recurrence (**C**) and FMT models (**D**). Results shown as mean log_10_ cfu/g wet biofilm mass from two biological replicates and at least four support structures. Error bars represent the standard deviation.

We characterised the biofilm communities using 16Sv4 rRNA gene sequencing to investigate the effect of antibiotics on the sessile community, and whether the biofilms in our experiments could be a source of both transient *C. difficile* spore detection post FMT and the origin of the recurrent CDI observed in the rCDI model. Taxonomic analysis and visualisation of the biofilm community isolated from these support structures highlight a varied community enclosed in an extracellular matrix forming a complex structure (Supplementary Fig. 2)^28^. Bifidobacteriaceae, Lactobacillaceae and Eubacteriaceae were the most abundant bacterial families present in the biofilm community prior to antibiotic exposure (Fig. 2). Post clindamycin exposure, an increase in the relative abundances of Enterobacteriaceae, Bacteroidaceae and Methanobacteriaceae were observed, which was accompanied by the decreased relative abundances of Bifidobacteriaceae and Eubacteriaceae. Vancomycin exposure, with no further intervention, was associated with the reduction in the abundance of several bacterial families; Bacteroidaceae, Eubacteriaceae, Lachnospiraceae, Ruminoccocaceae and Comamonadaceae had lower abundances for the remainder of the experiment compared with their pre-antibiotic abundance (Fig. 2A). FMT instillation was associated with the recovery of these same bacterial families at either 2- or 3-week post FMT, except for Comamonadaceae which did not recover by the end of the experiment (Fig. 2B). Furthermore, by direct enumeration, we recovered several different yeast species as part of the biofilm microbiota from both models, albeit at low levels.

Upon the addition of *C. difficile* into the lumen of the model, the bacterial spores became intimately associated with the biofilm structures present in all three vessels. During clindamycin induction and at peak CDI, the overall *C. difficile* levels recovered from the biofilm slightly decreased; however, the recovered *C. difficile* population was a mix of both spore cells and vegetative cells, ~1:3 ratio respectively. Sessile *C. difficile* cells accounted for approximately 0.007% of the total bacteria present in the biofilm community (Supplementary Fig. 3). Vancomycin therapy alone did not affect the recovery of *C. difficile* associated with the biofilm (Fig. 2C), nor was the instillation of FMT able to displace biofilm-associated *C. difficile* cells entirely (Fig. 2D).

### Biofilm-associated C. difficile cells can cause simulated CDI

**Fig. 3:**
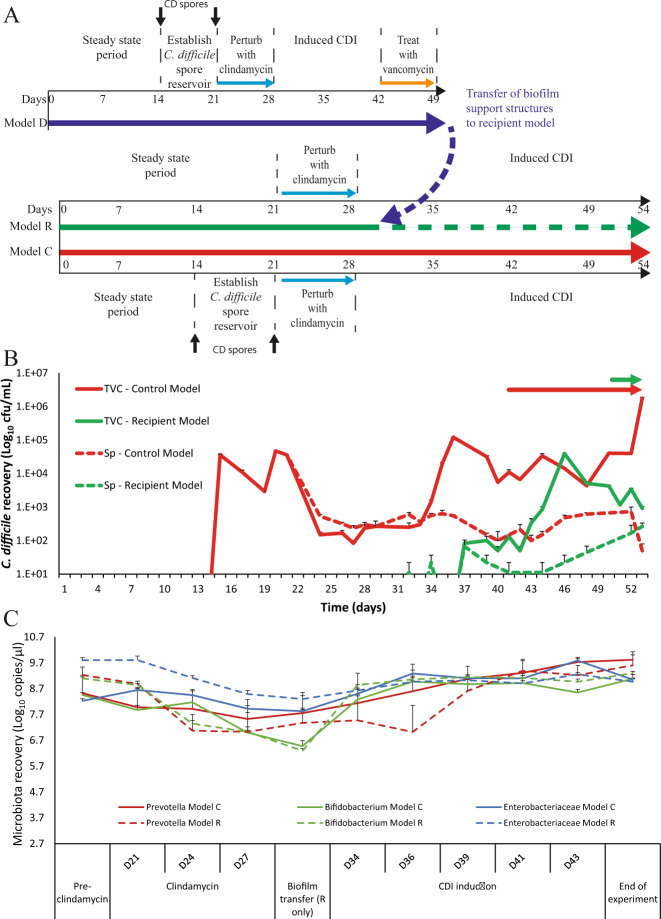
Contribution of biofilm-associated *C. difficile* to cause recurrent disease. **A** Timeline of events from the biofilm donor model (Model D, blue arrow) prior to support structure transfer, and two further models; a control model (Model C, red arrow) inoculated with *C. difficile* spores, and a biofilm recipient model (Model R, green arrow), which did not receive spores, but received the biofilm support structures from model D (broken blue arrow). **B** Recoveries of luminal *C. difficile* (total viable counts – TVC; spores - Sp) from model C (red line) and model R (green line) and subsequent periods of toxin detection (arrows). Solid lines are *C. difficile* total viable cells and broken lines are spore recoveries. Results expressed as mean log_10_ cfu/mL from two biological replicates. **C** Quantitative PCR enumeration of selected luminal microbiota populations from model C (whole lines) and model R (dotted lines) immediately prior to clindamycin instillation (pre-clindamycin), during clindamycin therapy and throughout CDI development and progression. Results expressed as mean log_10_ copy number per µL of luminal fluid from two biological replicates. All error bars represent the standard deviation.

Determining the role of biofilms in recurrent CDI has been particularly challenging with other in vitro and in vivo models of CDI as it has been difficult to independently delineate the luminal and planktonic populations. However, our model is ideally placed to investigate this question due to the accessibility of the biofilm support structures in our system. Here, we set up a biofilm transfer experiment, where a biofilm donor model (model D) underwent vancomycin ‘treatment’ of simulated CDI and the biofilm support structures from this model were transferred to a *C. difficile*-naïve recipient model (model R) and two independent biological replicates were performed (Fig. 3A). CDI was induced in model D following clindamycin exposure and at peak CDI, where *C. difficile* luminal recovery was 5.4 log_10_ cfu/mL (peak toxin was detected at 3 log_10_ reciprocal titre), vancomycin was instilled. Vancomycin treatment depleted the luminal-associated *C. difficile* population in the donor model to below the limit of detection (Supplementary Fig. 4AB) but, crucially, the biofilm-associated population remained present mostly as spores, as the vegetative cells were reduced (Supplementary Fig. 4CD). These biofilm support structures were then transferred to the recipient model. The mean *C. difficile* titre in the biofilm was assessed from two support structures at 3.8 log_10_ cfu/g wet biomass (Supplementary Fig. 4D). From this we estimate that a total of 4.1 log_10_ cfu *C. difficile* cells were transferred to the recipient model based on the number of support structures transferred and the average biofilm mass attached to each structure. The biofilm recipient model was exposed to clindamycin to create an environment conducive for CDI prior to the transfer of the support structures. Post biofilm transfer, luminal-associated *C. difficile* vegetative cells were recovered 9 days post transfer and toxin production was detected by the end of the experiment at 1 log_10_ reciprocal titre (Fig. 3B, green lines/arrow).

In parallel to the recipient model, we ran an experimental control model (model C). The purposes of this model were to ensure colonisation resistance had established in both R and C models, and that clindamycin exposure was able to create the microbial niche needed for CDI progression (Supplementary Fig. 3AB). Colonisation resistance was confirmed when the *C. difficile* spore dose added to the control model did not show spore germination or outgrowth (Fig. 3B). To confirm a CDI susceptible niche, clindamycin was instilled into the control model, alongside another inoculum of *C. difficile* spores and evidence of spore germination was detected 7 days post clindamycin, followed by vegetative outgrowth and toxin production, detected from day 41 onwards and at a peak of 3.0 log_10_ reciprocal titre (Fig. 3B, red lines/arrow).

We monitored the microbiota dynamics and the effects of clindamycin within the control and recipient models. The microbial populations enumerated by quantitative PCR from both models were similar immediately prior to antibiotic instillation (Fig. 3C and Supplementary Fig. 5). Clindamycin had a pleiotropic effect on the microbiota, causing an average decrease of at least 1 log_10_ copies/µL in *Prevotella* spp., *Bifidobacterium* spp. and *Bacteroides* spp., whilst Enterobacteriaceae and *Enterococcus* spp. increased by at least 1 log_10_ copies/µL in all models. The monitored microbiota recovered to pre-clindamycin levels by day 43.

### Biofilm microbiota can affect *C. difficile* biofilm formation

Following the findings that *C. difficile* cells associated with the biofilm were unaffected by either antibiotic therapy or FMT microbial therapy, having the potential to cause disease, we investigated the influence of other microbes on *C. difficile* biofilm formation in vitro. Microorganisms were cultured directly from the biofilm support structures in our gut model and identified to the species level by MALDI-TOF analysis (Supplementary Table 1). These biofilm isolates were co-cultured with *C. difficile* and the effect on *C. difficile* biofilm formation was characterised as either antagonistic (the isolate reduced *C. difficile* biofilm formation), co-operative (summation of individual mono-species biomass is equal to that of the co-culture biofilm) or synergistic (the isolate enhanced *C. difficile* biofilm formation).

**Fig. 4:**
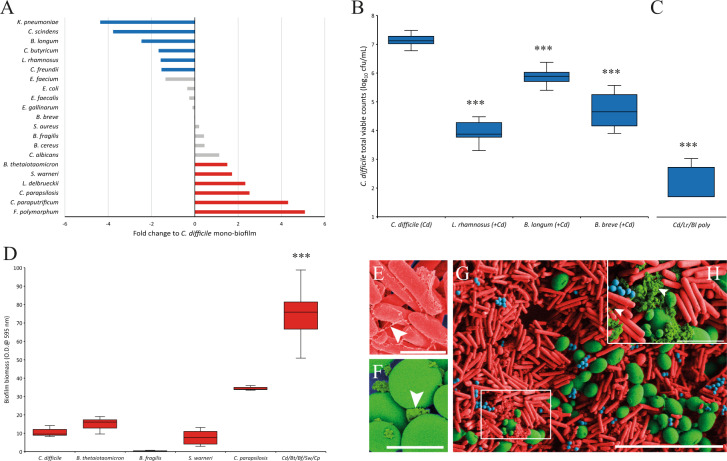
Biofilm-associated microbiota can affect *C. difficile* biofilm formation. **A** Biofilm formation of *C. difficile* when co-cultured with different microbial species, compared with the biofilm formed from a *C. difficile* monoculture. Blue or red bars indicate species that significantly (*p* ≤ 0.05) reduced or increased the biofilm formed and grey bars represent species that did not significantly affect biofilm formation. Results expressed as fold change of crystal violet absorption of the dual co-culture vs monoculture. **B** Antagonistic bacteria reduced *C. difficile* recoveries from biofilms. *C. difficile* recoveries from mono and dual culture biofilms and **C** the enhanced antagonistic effect of polymicrobial biofilms on *C. difficile* recoveries from biofilms. **D** Poly synergistic biofilms are greater than the sum of the individual monoculture biofilms. **B**–**D** Results expressed as box and whisker plots showing the median log_10_ cfu/mL, lower and upper quartile ranges, and the minimum and maximum results from at least four technical replicates from three biological repeats. False-coloured SEM of *C. difficile* (**E**, ×10,000, scale bar is 2 µm) and *C. parapsilosis* (**F**, ×5000, scale bar 5 µm) monoculture biofilms. Polymicrobial biofilms of *C. difficile* (red cells), *S. warneri* (blue cells) and *C. parapsilosis* (green cells) showing close interaction between the microbial cells (**G**, ×2500, scale bar 20 µm). Insert, zoomed section highlighting the extracellular matrix-like substance (light green colour) (**H**, ×10,000, scale bar 2 µm). White arrows denote what appears to be extracellular matrix in all images. Asterix denotes significantly difference with a *p* value < 0.01.

A wide range of bacterial and yeast species were identified associated with the biofilm support structures (Supplementary Table 1) removed at different time points throughout the recurrence and FMT gut models. Initially, these microbial species were individually co-cultured with *C. difficile*, where six microbial species were found to act antagonistically to significantly (*p* ≤ 0.05) reduce the biofilm biomass produced and four microbial species acted in a synergistic manner to significantly (*p* ≤ 0.05) increase the biomass produced in these biofilms (Fig. 4A and Supplementary Fig. 6). Additionally, two microbial species, *Lactobacillus delbrueckii* and *Clostridium paraputrificum*, were identified as co-operative species, as the sum of the biomass from individual biofilms was equal to that of the dual species’ biofilms with *C. difficile*. We determined if the reduced co-culture biomass from those ‘antagonistic’ species against *C. difficile* was a result of decreased biofilm matrix production or a reduced number of *C. difficile* cells within the biofilm. Direct enumeration of the *C. difficile* viable cells from co-culture biofilms showed significantly less *C. difficile* cells compared with monoculture biofilms (Fig. 4B). Interestingly, co-culture of *C. difficile* with *Bifidobacterium breve* also reduced the number of *C. difficile* biofilm cells, even though there was no significant difference in the biomass (Fig. 4AB). *Lactobacillus rhamnosus*, *Bifidobacterium longum* and *B. breve* all had a reductive effect of 3.3, 1.2 and 2.5 log_10_ cfu/mL, respectively, on *C. difficile* counts. These reductions in *C. difficile* cells within the biofilm coincided with reduced toxin activity detected, ~2 log_10_ reduction in toxin titre (from 3.5 to 1.5 log_10_ median toxin titre), in the medium (data not shown). Co-culturing *L. rhamnosus* and *B. longum* with *C. difficile* in a polymicrobial biofilm caused an additive antagonistic effect on the *C. difficile* biofilm formation, where a reduction of 4.4 log_10_ cfu/mL was seen (Fig. 4C).

Of those microbial species that were able to enhance *C. difficile* biomass in dual cultures, none increased the number of *C. difficile* cells within the biofilm; however, they all increased the amount of biofilm biomass produced in dual co-culture biofilms (Supplementary Fig. 7C). Interestingly, when several of these microbial species were cultured together in a polymicrobial biofilm, the resulting biofilm biomass was greater than the sum of the individual monocultures (Fig. 4D). Scanning electron microscopic imaging of either *C. difficile* or *Candida parapsilosis* monoculture biofilms showed what appears to be the extracellular matrix produced by either species with distinctive physical characteristics. *C. difficile* produces a filamentous-like matrix (Fig. 4E)^12,13^ whereas *C. parapsilosis* produces a dense granular extracellular matrix (Fig. 4F)^30^; however, we did not determine the composition of the specific extracellular matrix from each species. In a polymicrobial biofilm of *C. difficile*, *C. parapsilosis* and *Staphylococcus warneri*, the individual microbial cells showed a close interaction with each other in a heterogeneous biofilm with the produced species-specific extracellular matrix-like substance encompassing the microbial cells of the different species present (Fig. 4GH). This combination of microbial species in a polymicrobial biofilm did not result in an increased recovery of the individual microbial species, rather it appears that the increase in biofilm biomass was due to an observed increase in extracellular matrix-like substance and cell debris (Supplementary Fig. 7).

We further characterised the interactions of *C. difficile* and the sessile microbial community in polymicrobial biofilms where microbial species with both an antagonistic and a co-operative or synergistic behaviour towards *C. difficile* were included. Polymicrobial biofilms containing *C. albicans* were able to abolish the antagonistic effect of *L. rhamnosus* on *C. difficile* biofilm formation, despite a 2.5 log_10_ cfu/mL increase in *L. rhamnosus* cells in the polymicrobial biofilm, as *C. difficile* recoveries were similar to those from a monoculture biofilm (Supplementary Fig. 8A). However, in polymicrobial biofilms of *C. difficile*, *Bacteroides thetaiotaomicron* and *B. longum*, the antagonistic effect of *B. longum* on *C. difficile* biofilm formation was further exacerbated (Supplementary Fig. 8B). A reduction in *C. difficile* recoveries of 1.2 log_10_ cfu/mL, compared with monoculture recoveries, were observed in *C. difficile/B. longum* co-culture experiments (Fig. 4B); however, in *C. difficile/B. thetaiotaomicron/B. longum* polymicrobial biofilms, *C. difficile* recoveries were reduced by 2.3 log_10_ cfu/mL, compared with monoculture recoveries, (Supplementary Fig. 8B).

## Discussion

Failed CDI therapies notably lead to recurrent infections with increased morbidity and mortality^1,3,7,31^. With the chance of further recurrent episodes increasing dramatically after each failed therapy, it appears that *C. difficile* can occupy a niche whereby it is protected from antimicrobial assault. The ability of *C. difficile* to form a biofilm in vitro^11,12,16^ and form a mono-species biofilm in vivo^20^ has been demonstrated. Furthermore, in vivo studies have shown that *C. difficile* cells can associate with the microbial communities found within mucosal biofilms^18–20^. The compact nature, microbial cells’ metabolic state^32^ and the surrounding extracellular matrix of biofilms can reduce the egress of antimicrobials into the biofilm and affect their efficacy. It has been hypothesised that biofilms represent a niche that can be occupied by *C. difficile* where it is protected from the effects of antibiotics.

Here we used a clinically reflective human colon model to simulate recurrent CDI and the efficacy of FMT treatments, simulating the clinical outcome of this treatment, and to elucidate the role of biofilms in recurrent disease. This model provides a valuable opportunity to separately delineate the contributions of both the biofilm and luminal microbial populations towards recurrent CDI. Vancomycin treatment in our model successfully depleted the luminal *C. difficile* populations; however, without FMT instillation, *C. difficile* recurrence occurred within 30 days, similar with a clinical setting^29^. Whilst FMT instillation was associated with prevention of recurrent CDI, we detected transient luminal *C. difficile* spores post-FMT. Analysing the biofilm microbial populations throughout these models highlighted that, upon exposure, *C. difficile* spores were able to associate with the biofilm, and, after germination, spores and vegetative cells were recovered from the biofilm until the end of the experiment. The exosporium layer of ribotype 027 spores is ~110-nm thick with a hair-like nap that is proposed to contribute towards adhesion of *C. difficile* spores to surfaces, potentially including the extracellular matrix found embedding biofilms^33^. The impact of vancomycin treatment on biofilm populations reduced the abundance of several bacterial families, whereas, instillation of an FMT restored some of these populations at an earlier time point compared with vancomycin alone treatment. This suggests that FMT treatment was able to replenish the biofilm microbiota after antibiotic induced depletion. In our studies, we found that neither vancomycin nor FMT successfully depleted the biofilm-associated *C. difficile* populations, thus leaving this potential source of *C. difficile* intact. Indeed, antimicrobials have displayed reduced efficacy against *C. difficile* biofilms^34^ and have actually been shown to induce biofilm formation^13,14^.

We have shown the capacity of these biofilm-associated *C. difficile* populations to populate the luminal space and produce toxin, potentially causing disease, given a susceptible environment. At the time of biofilm transfer, the sessile *C. difficile* cells appeared as spores after vancomycin exposure, which indicates that germinating spores seeded the planktonic phase, rather than dissemination of vegetative cells. During *C. difficile* luminal proliferation in the recipient model, the microbiota were recovering to pre-clindamycin levels, which could have limited the extent of CDI in this experiment. The amount of biofilm biomass transferred from the donor model to the recipient model was estimated to harbour ~4.1 log_10_ cfu *C. difficile* cells. The extensive amount of biofilm found within the human proximal colon^35^ means that potentially higher levels of *C. difficile* within the in vivo proximal colon biofilm could lead to a more severe disease phenotype. Here we show that multispecies biofilms formed in an in vitro model can harbour and protect *C. difficile* from antimicrobial therapy and FMT installation, and can contribute towards recurrent CDI. Research on the long-term outcomes of patients following FMT therapy reported that between 8 and 18% of FMT patients suffered a recurrent episode of CDI^36,37^. In each study the authors reported a high number (75%) of these recurrence cases were attributable to post-FMT antibiotic prescription, some of which are not implicated as CDI inducing antibiotics, i.e. penicillin^37^. Thus, the presence of *C. difficile* within biofilms could have clinical implications for future patient antibiotic prescription management post-FMT.

Given the importance of the biofilm community, we investigated the interaction between different biofilm species towards *C. difficile* biofilm formation. We observed several synergistic microbial species, namely *C. parapsilosis, S. warneri* and *B. thetaiotaomicron*, which enhanced the biofilm biomass and had a close interaction with *C. difficile*. The interaction of these microbial species with *C. difficile* can have surprising effects, i.e. when grown together, *Candida* spp. sustains the growth of *C. difficile* under aerobic, normally toxic, conditions^15^. Additionally, *B. thetaiotaomicron* secretes sialidases to release sialic acids from host mucus and also produces the metabolic product succinate; both succinate and sialic acids are utilised by *C. difficile* during expansion during disease^38,39^. The close proximity of *C. difficile* and *B. thetaiotaomicron* cells in a biofilm are ideal conditions for *C. difficile* to exploit these metabolic nutrients. Donelli, Vuotto^11^ observed a synergistic interaction in the biofilm formation of *C. difficile* and *Finegoldia magna*, where the extracellular matrix produced entangled both organisms. However, some bacteria had an antagonistic effect on *C. difficile* biofilm formation, namely *L. rhamnosus* and *Bifidobacterium* spp. *Lactobacillus* spp. and *Bifidobacterium* spp. are known to secrete organic acids into the extracellular media and the acidification of the environment could affect *C. difficile* biofilm formation^40^. In support of this hypothesis, we observed a decrease in biofilm formation of *C. difficile* monocultures when the growth media was acidified to pH 5. Biofilm-associated Lactobacillaceae and Bifidobacteraceae were reduced after exposure to antibiotics in our model, thus reducing the antagonistic effect of the members of these families on *C. difficile* biofilm formation. Biofilms formed by different *Lactobacillus* spp. are known to reduce contamination of other pathogenic bacteria^41^ and members of Lactobacillaceae and Bifidobacteraceae are often used in probiotics/microbial therapeutic cocktails to resolve recurrent CDI^40,42–44^. However, the interplay between sessile organisms is malleable depending on the other organisms present in the biofilm. For instance, the antagonistic effect of *L. rhamnosus* towards *C. difficile* biofilm formation can be alleviated in the presence of *C. albicans*. In contrast, the antagonistic effect of *B. longum* was exacerbated with the addition of *B. thetaiotaomicron*. Our data suggests that the biogeography of the sessile microbiota upon *C. difficile* infiltration can impact upon the biofilm formation of *C. difficile*, potentially enhancing *C. difficile* growth or providing a less favourable growth environment. A note of caution is needed when extrapolating these findings to an in vivo setting as these interactions can be condition or microbial strain dependent.

Data from in vitro and in vivo models show the capacity of *C. difficile* to form a mucosal biofilm and co-localise with different sessile microbes encased in an extracellular matrix-like substance. Here we show that biofilms formed by gut-derived microbial communities can act as a reservoir for *C. difficile* with the potential to cause recurrent disease, and these biofilm-associated populations remain unaffected by either antibiotic treatment or microbial replacement therapy. Taken together, these data can provide an explanation for antibiotic failures in CDI patients, and possibly the source of recurrence in FMT patients following subsequent antibiotic exposure. Our data highlight the need to test the efficacy of novel therapeutics on both the luminal and biofilm populations to ensure effective CDI treatments. However, the mechanisms behind the interactions between *C. difficile* and other biofilm microbiota require further research.

## Methods

### Strains used in this study and growth conditions

Two *C. difficile* strains were used in this study; strain 210 (BI/NAP1/PCR ribotype 027/toxinotype III) was originally isolated in 2005 during an outbreak at the Maine Medical Centre (Portland, ME, U.S.) and used in all of the gut model experiments, strain R20291 (BI/NAP1/PCR ribotype 027/toxinotype III) was originally isolated in 2004 during an outbreak at the Stoke Mandeville Hospital (Stoke, U.K.) and used in the co-culture experiments. *C. difficile* strains were grown either on CCEYL agar plates or in BHI broth supplemented with yeast extract (5 g/L) and l-cysteine (0.25 g/L) (BHISC) incubated anaerobically at 37 °C for 18–48 h. Strains used in the co-culture experiments were grown on Columbia blood agar plates (E&O Laboratories, U.K.), either anaerobically or aerobically (depending on the organism) at 37 °C.

### Ethics and in vitro gut model set up

The collection and use of human faeces in our gut model has been approved by the School of Medicine Research Ethics Committee, University of Leeds (MREC 15-070–Investigation of the Interplay between Commensal Intestinal Organisms and Pathogenic Bacteria). The assembly of triple-stage chemostat gut models to simulate CDI and recurrence is described here^23,24,28^. Briefly, for each model, three glass vessels were arranged as shown in Supplementary Fig. 1, and maintained at 37 °C, under anaerobic conditions and pH controlled to represent the proximal (vessel 1, 280 mL void, pH 5.5), medial (vessel 2, 300 mL void, pH 6.2) and distal colon (vessel 3, 300 ml void, pH 6.7). A complex growth medium^28^ was top fed into vessel 1 at a rate of 0.015 L/h^−1^. The microbial abundance in the vessels has been previously validated against the intestinal contents of sudden death victims and it provides a close simulation of microbial activities and composition to the different areas of the human colon^45^.

### Gut model timeline

The experimental timeline for each experiment is described in the separate figures, but here is a general timeline for the gut model experiments; results from each gut model experiment are from at least two biological replicates. Each vessel of each model is inoculated with 160 mL of 10% w/v pooled faecal slurry, diluted with pre-reduced sterile PBS, from five healthy CDI negative donors. All donors were anonymous, ≥60 years of age and with no history of antibiotic therapy for the previous three months. Each faecal sample was checked for the presence of *C. difficile* glutamine dehydrogenase (GDH) using the EIA C. DIFF CHEK^TM^ test (Tech Lab, U.S.). Microbial populations were allowed to reach equilibrate growth before a single dose of ~10^7^ cfu/mL *C. difficile* strain 210 spores (prepared following the method of Buckley, Spencer^17^) were added to vessel 1 of each model. This was done to establish that the microbiota had formed colonisation resistance against *C. difficile* germination. One week later, another dose of *C. difficile* spores was added to the model and the microbiota were disrupted with clindamycin (dosed at 33.9 mg/L, four times daily for seven days^26^). Thereafter, *C. difficile* recoveries were enumerated daily for germination, outgrowth and toxin production (simulated CDI). At peak toxin production, vancomycin ‘treatment’ was instilled (dosed at 125 mg/L, four times daily for seven days^26^). Simulated recurrent CDI was monitored up to 35 days post vancomycin.

### Faecal microbiota transplant instillation

A faecal sample from a single donor was screened for the presence of *C. difficile* as previously described. A 10% w/v slurry was made by diluting the faecal sample with pre-reduced sterile PBS and 50 mL was instilled into vessel 1 at a rate of 50 mL/h (as shown in Supplementary Fig. 1), 3 days post vancomycin cessation. The tubing was washed through with an extra 30 mL pre-reduced sterile PBS.

### Biofilm support structure sampling

Biofilm support structures were screwed into the lid of vessel 3 during model assembly. At selected time points, three rods were removed from the lid and transferred to 5 mL pre-reduced PBS and vortexed. The rod was removed, 2 mL of this fluid was centrifuged, and the microbial pellet weighed and used for DNA extraction. The remainder of the fluid was used to enumerate the microbial populations on selective and non-selective media (described below) and reported as mean log_10_ cfu/g wet biofilm biomass.

### Microbiota enumeration, isolation and identification

Luminal and biofilm culture fluid were serially diluted in pre-reduced peptone water and 20 µL of each dilution was inoculated on to different agar plates and incubated as outlined in Supplementary Table 2. Microbial colonies were enumerated and identified based on colony morphology and MALDI-TOF identification. Identified colonies were sub-cultured onto non-selective plates for purity, stored in glycerol broth (10% v/v) and kept at −80 °C.

### DNA extraction of luminal and biofilm samples

Luminal gut model fluid was collected from vessel 3 of control and recipient models at each sampling point. One DNA extraction was performed from each biofilm support structure. Total DNA from luminal or biofilm samples was extracted using FastDNA^TM^ SPIN kit for soil (MP Biomedicals^TM^, U.K.) following manufacturer’s instructions with DNA stored at −80 °C.

### Quantitative PCR

Each sample was quantified using a Nanodrop 2000c and normalised to 5 ng/µL. Levels of bacterial genus/species of the human microbiota were determined by quantitative PCR using the primers and conditions previously described^46^. For each microbial group, 15 μL reactions containing final concentration of SYBR Green 1x Master Mix (Qiagen, U.K.), 0.3 μM primers, and 18.75 ng of DNA template were prepared. The Eubacteria test also included a FAM-tagged probe at 0.25 μM. Reactions were analysed in a Rotor-Gene Q (Qiagen, U.K.). Each DNA extract was analysed in triplicate alongside plasmid DNA standard curves ranging from 5 × 10^9^ copies/µL to 500 copies/µL^46^. The change in bacterial levels were converted to logarithms of 16S rRNA gene copy numbers to achieve normal distribution.

### Cytotoxin assay

Luminal aliquots were centrifuged at 16,000×*g* for 10 mins and filtered using a 0.22-µm filter unit and applied to cultured Vero cells as previously described^17^. Briefly, serial dilutions of filtered fluid were applied to a monolayer of cultured cells. *C. sordellii* toxin antisera (Prolab, U.K.) was added as a neutralising control to one well of each sample. Trays were incubated at 37 °C 5% CO_2_ for 48 h. Cytotoxin titres were correlated to an arbitrary log_10_ scale and expressed as relative units (RUs) at the highest dilution, with >70% cell rounding (i.e., 10^0^, 1RU; 10^-1^, 2RUs; and 10^-2^, 3RUs). The limit of detection was 1 RU for toxin titre.

### Antibiotic concentration determination

Antibiotic concentrations in for each model were determined by microbiological bioassay, as previously described^26^. Briefly, indicator organisms *Kocuria rhizophila* (ATCC 9341, clindamycin) and *Staphylococcus aureus* (ATCC 29213, vancomycin) were inoculated into Wilkins-Chalgren agar or Mueller-Hinton agar, respectively, and poured into 245 × 245 mm agar plates. Once set, 25 wells were generated and 20 µL of antibiotic calibrator or filtered gut model fluid were added to each well. Plates were incubated at 37 °C for 24 h after which the inhibition zone diameters were measured. Unknown concentrations from gut model samples were determined from the calibration curve. All assays were performed in triplicate.

### Bacterial 16S rRNA library preparation and sequencing

To characterise biofilm communities, 16S rRNA gene V4 sequences were PCR-amplified from 1 μl of DNA extract using the AccuPrime High Fidelity PCR kit (Invitrogen Catalog No 12346094) with the primer pair 515F (5′ AGCMGCCGCGGTAA 3′) and 806R (5′ GGACTACHVGGGTWTCTAAT ′3) containing Illumina MiSeq adaptors and single-end barcodes. PCR temperature cycles weres: 98 °C for 3 s, 33 cycles of: 98 °C for 20 s, 50 °C for 30 s, 72 °C for 90 s; then 72 °C for a final 10 min. Amplicons were pooled in equal quantities, cleaned with AMPure beads (Beckman Coulter) and paired-end sequenced on the MiSeq platform following Nextera XT library preparation (Illumina).

### Taxonomic assignments

Reads were demultiplexed with the split_libraries_fastq.py function in Qiime (version 1.9.1)^47^ and identical sequences were binned into amplicon sequence variants (ASVs) using the programme DADA2 (version 1.4.0, parameters EE = 2, TruncL = c(200, 180) and *q* = 10)^48^. The assign Taxonomy function in DADA2 was used to assign a taxonomic name to each unique ASV using the RDP Classifier with the SILVA 16S rRNA database (Silva nr v128)^49,50^. Low abundance reads (≤10 reads) were removed from further analysis. Reads for each sample were aggregated to the family taxonomic level and converted to percentage abundance. Results shown are the mean abundance from at least three biofilm support structures from each model. Bacterial families shown in Fig. 2 represent all families whose values were ≥1% abundance at a single sampling point throughout the model timeline; the values from other bacterial families where the abundance was ≤1% were aggregated and labelled as ‘other’.

### In vitro biofilm assay

We used a modified version of the in vitro biofilm assay by Dawson, Valiente^12^. All microorganisms used in this assay were isolated from biofilms produced from these gut models. Sterile 13 mm glass coverslips were inserted into the bottom of 24-well plates and 1.8 mL of sterile pre-reduced BHISC broth was added to each well. To these wells, 200 µL of the overnight cultures of organisms to be tested were added; on each plate, four wells of each monoculture biofilms were setup and four wells of co-culture biofilms. An uninoculated negative control was also setup on each plate. Plates were incubated anaerobically at 37 °C for 3 days without agitation. Glass coverslips were removed and thrice washed in sterile pre-reduced PBS and used for either the quantitative crystal violet biofilm biomass assay or to determine the total viable counts.

### Measuring the biofilm biomass using crystal violet

Washed coverslips were incubated with 500 µL of filtered 1% crystal violet solution for 30 min. Coverslips were washed twice in sterile PBS and further incubated with 500 µL 100% methanol for 30 mins. Biofilms were disrupted by vigorous pipetting, samples serially diluted in PBS and the optical density of each sample measured at 595 nm (Infinite P200 Pro, Tecan). The glass coverslip from the negative control wells were used as a blank. The *C. difficile* monoculture biofilm biomass was compared with polymicrobial cultures and results are expressed as fold change from at least three biological replicates and three technical replicates from each biological replicate.

### Enumeration of total viable cells

This assay was done under anaerobic conditions. In all, 500 µL of sterile pre-reduced PBS was added to the washed glass coverslips and the biofilm disrupted with vigorous pipetting. Each sample was serially diluted and plated out onto selective agar as outlined above. Results are expressed as log_10_ cfu/mL from at least three biological replicates and three technical replicates from each biological replicate.

### Scanning electron microscopy

Biofilms were grown on glass coverslips, harvested after 3 days and rinsed twice in PBS. They were fixed in 2.5% glutaraldehyde in 0.1 M phosphate buffer overnight and washed twice in 0.1 M phosphate buffer for 30 min each. Biofilms were post fixed in 1% osmium tetroxide in phosphate buffer for 2 hours and washed twice for 20 mins in phosphate buffer before dehydrating using an ascending acetone series (20%, 40%, 60%, 80%, 100%). Biofilms were then critical point dried (Polaron E3000, Quorum Technologies) using liquid CO_2_ as the transition fluid. The biofilm samples were mounted on 13 mm diameter pin stubs and coated with platinum to a thickness of 5 nm (Cressington 208HR). Biofilms were imaged using a Hitachi SU8230 ultra high-resolution field emission scanning electron microscope (FE-SEM). Selected images were false-coloured using Adobe Photoshop CC version

### Statistical analysis and graphical software

Statistical analysis was performed using IBM SPSS Statistics 22 for Windows. Co-culture data were analysed using a Mann–Whitney *U* test. Polymicrobial biofilms and all co-cultures involving supernatants were analysed with the Kruskall–Wallis one-way analysis of variance with a pairwise comparison. *P* values of ≤0.05 were considered statistically significant. GraphPad Prism 5 for Windows, version 5.03 and Microsoft Excel 365 were used to generate figures.

### Reporting summary

Further information on research design is available in the Nature Research Reporting Summary linked to this article.

## Supplementary information

Supplementary Information

Reporting Summary

## Data Availability

The 16s rRNA taxonomic analysis generated and used during the current study are available in the Research Data Leeds Repository, University of Leeds, with a DOI (10.5518/784).
