# Supplementary material for: Biofilms harbour Clostridioides difficile, serving as a reservoir for recurrent infection
Source: NPJ Biofilms Microbiomes. 2021 Feb 5;7:16. doi: 10.1038/s41522-021-00184-w (PMC7864922; doi:10.1038/s41522-021-00184-w)
Supplement: Supplementary file 1 — Supplementary Information [file 41522_2021_184_MOESM1_ESM.pdf]

<u>BIOFILMS HARBOUR *CLOSTRIDIOIDES DIFFICILE*, SERVING AS A RESERVOIR FOR RECURRENT INFECTION: SUPPLEMENTARY MATERIAL</u>

Supplementary figure 1

***In vitro* gut model and faecal microbiota transplant set up.** (**A**) Three chemostat vessels are arranged in a weir-cascade fashion and top fed with a complex growth mixture. Each vessel mimics the physio-chemical conditions as you traverse the human colon (proximal, medial and distal colon). (**B**) FMT set up used in our experiments, simulating the nasal-jejunal route of administration used at Leeds General Infirmary. In this way, 50 mL of 10% w/v faecal slurry was instilled into the base of vessel 1 over one hr.

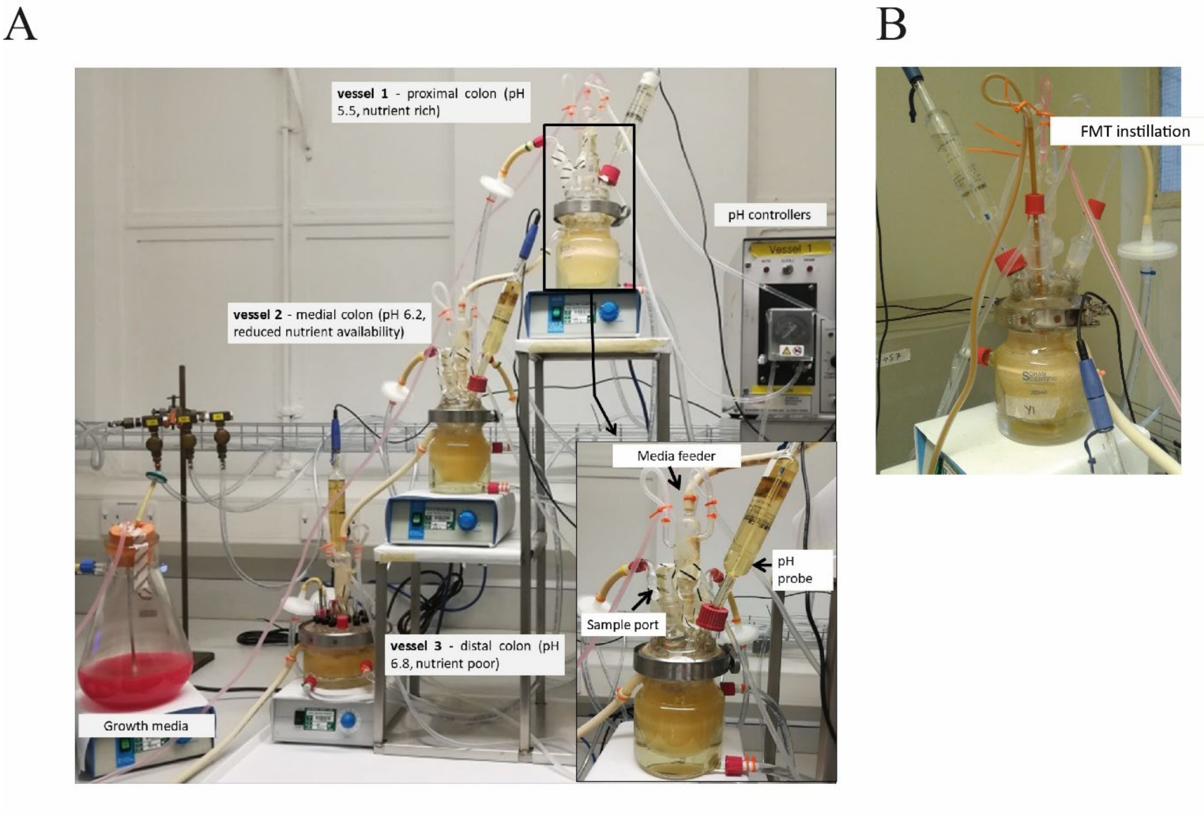

Supplementary figure 2

Biofilm support structures. (**A**) Glass etched rods were suspended from the vessel lid allowing easy removal of each rod. (**B**) Scanning electron microscopy of the biofilm formed on a rod after incubation within the gut model system. Image is at 95x magnification and scale bar is 100 µm. (**C**) SEM of the multispecies biofilm formed on the support structures. Image is at 16,600x magnification and black scale bar is 5 µm.

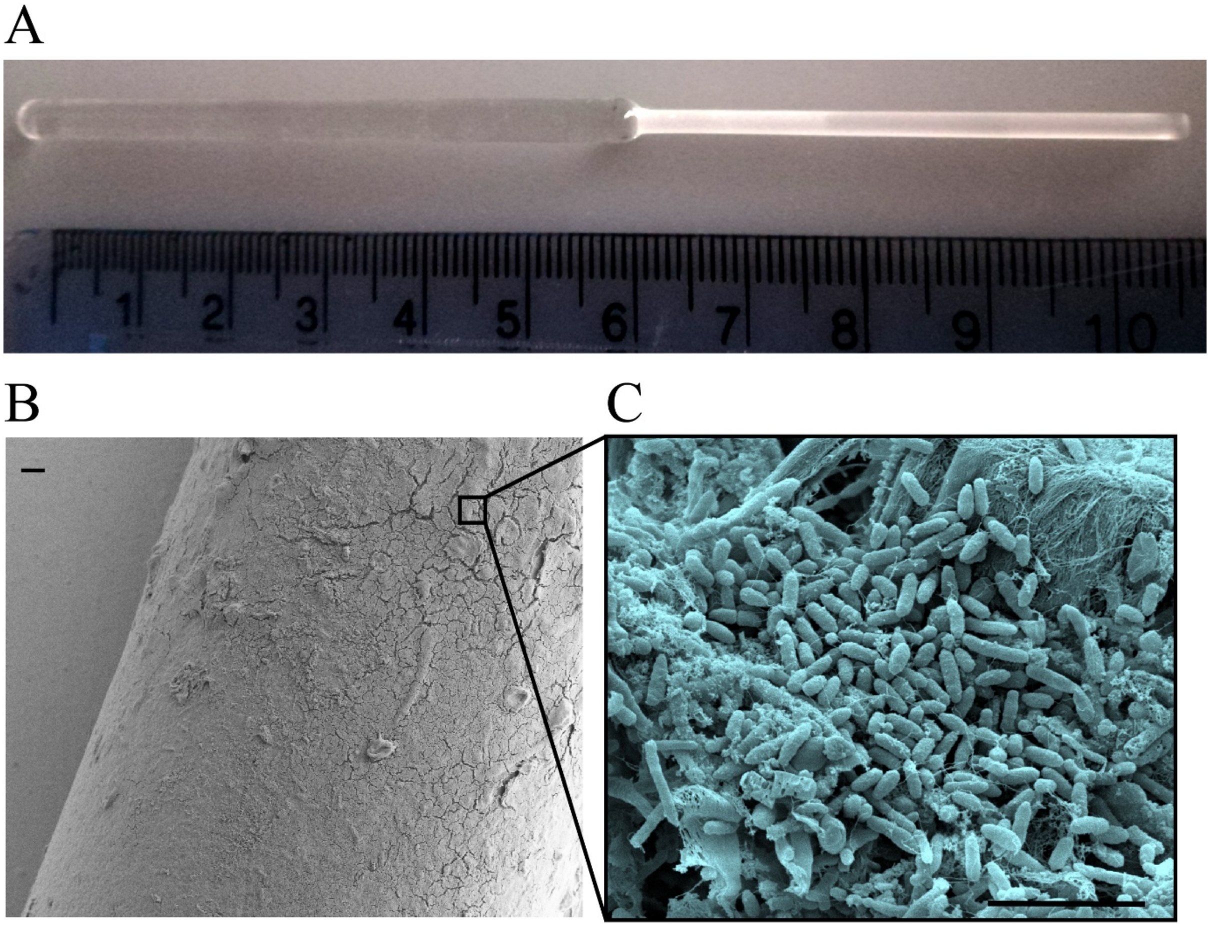

Supplementary figure 3

Quantitative PCR enumeration of the total sessile bacteria (Eubacteria) and sessile *C. difficile* recovered from the biofilm support structures. Results shown are mean copies/µl from two biological replicates (eight technical replicates in total).

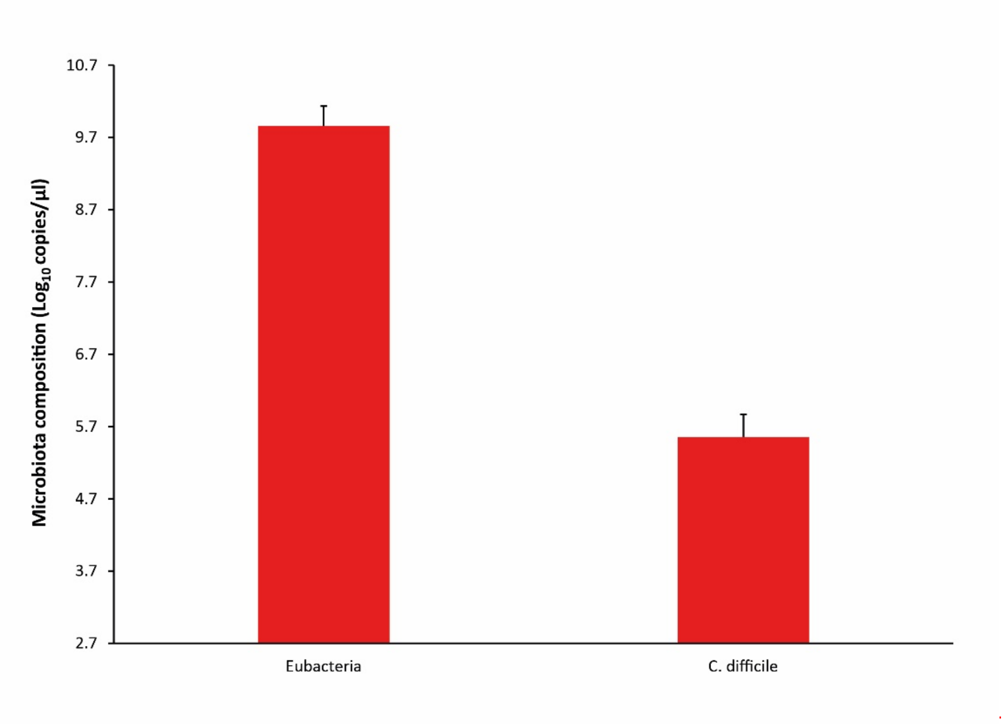

Supplementary figure 4

Model schematic and *C. difficile* recovery from the biofilm donor model (model D). (**A**) Timeline of events from the biofilm donor model prior to biofilm support structure transfer. Two doses of *C. difficile* spores were added prior to primary CDI. Simulated CDI was then treated with vancomycin reducing the luminal *C. difficile* populations to undetectable levels. (**B**) Recovery of *C. difficile* total viable counts (red line) and spores (blue line) from model D, and toxin detection (red arrow). Error bars represent the standard deviation from two biological replicates. (**C**) Recovery of sessile *C. difficile* vegetative (red bars) and spores (blue bars) from model D pre-vancomycin and (**D**) at time of transfer to recipient model. Error bars represent the standard deviation from six technical replicates from two biofilm support structures for each time point.

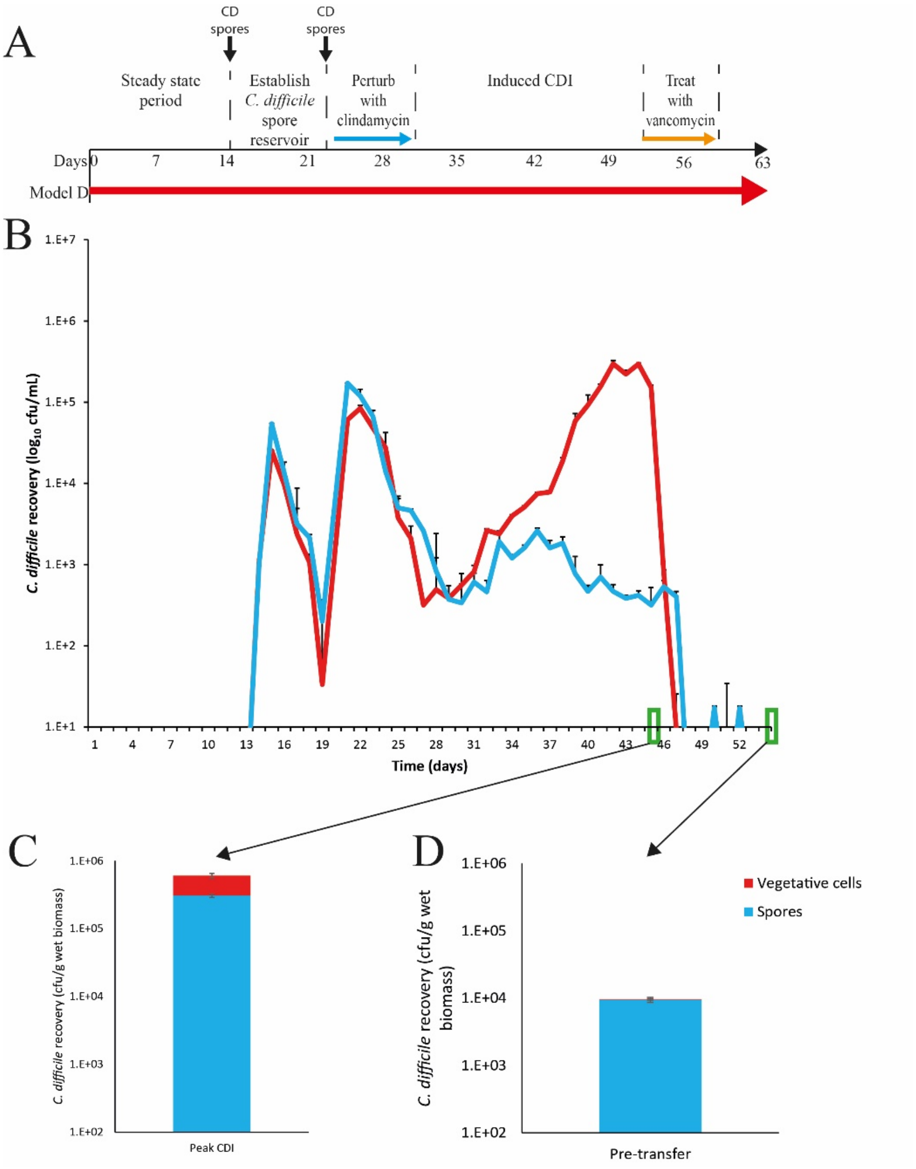

Supplementary figure 5

Quantitative PCR of luminal bacterial populations from control (A) and recipient (B) models. Biofilm populations were transplanted from model D to model R on day 30. Results expressed as mean $\log_{10}$ copy number per μL of luminal fluid from two biological replicates.

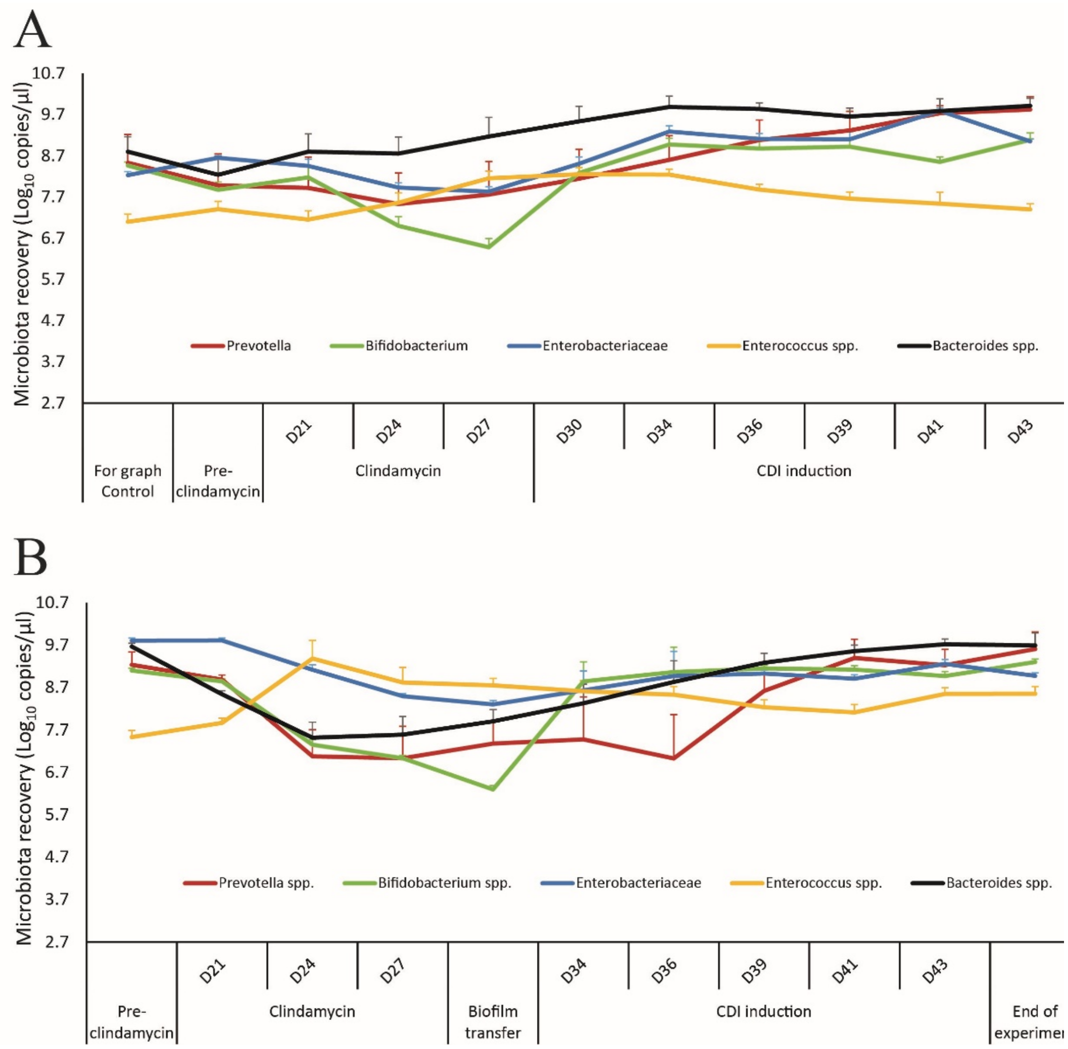

Supplementary figure 6

***In vitro* biofilm formation of *C. difficile* (red bar), different mono-cultured species (green bars) and dual biofilms with *C. difficile* (blue bars).** Results shown are mean crystal violet absorption from at least three biological replicates and 12 technical replicates. Error bars represent standard deviation.

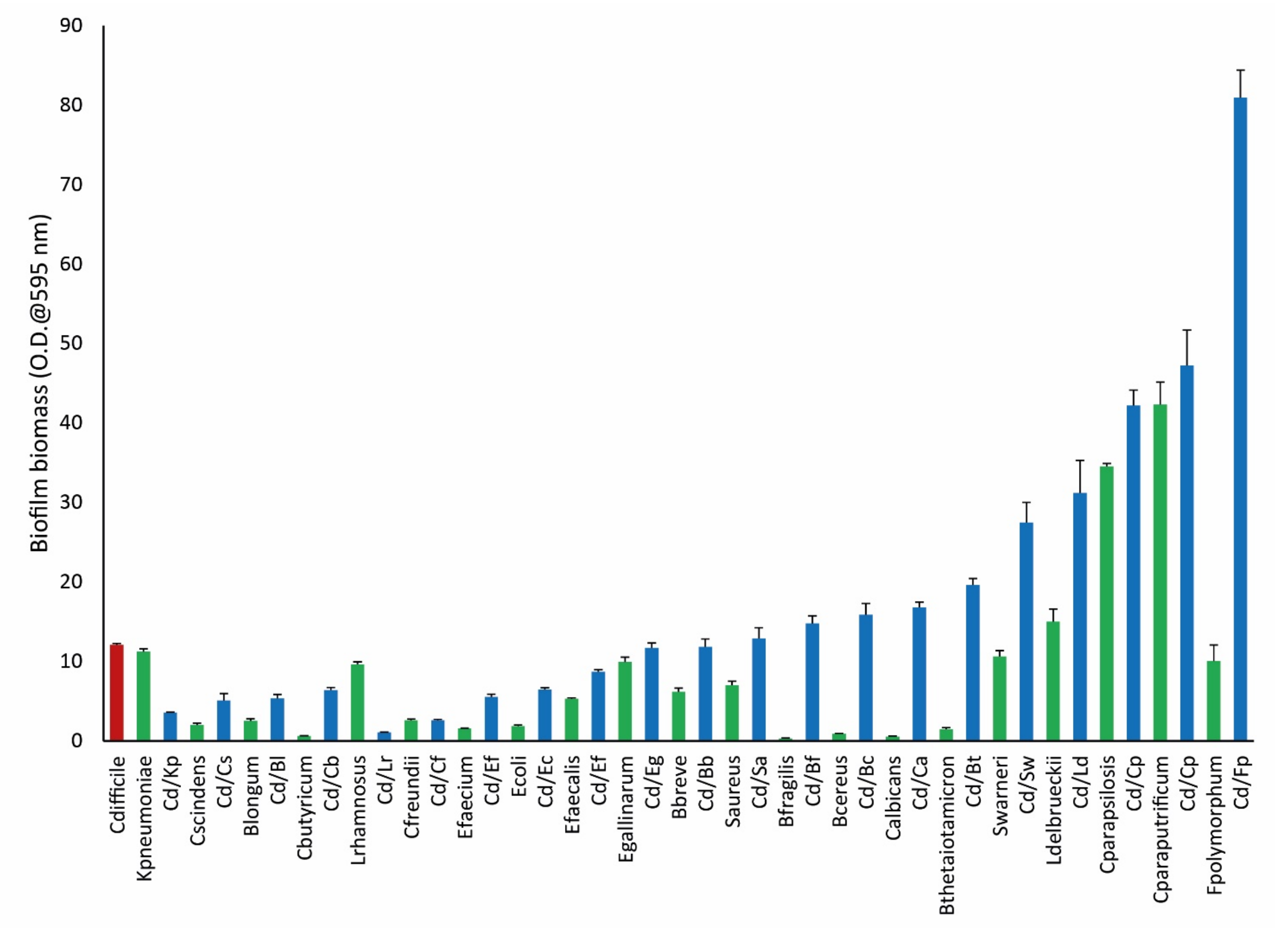

Supplementary figure 7

Monoculture biofilms and mixed as a polymicrobial biofilm as shown in Figure 4G. (A) Biomass as measured by crystal violet absorption of monoculture biofilms and polymicrobial biofilms. (B) Bacteria were enumerated using selective agars from monoculture (blue) and polymicrobial (orange) biofilms using selective agars (Supplementary Table 1). Results shown are from three biological replicates and at least 12 technical replicates. (C) SEM of the polymicrobial biofilm from (A & B).

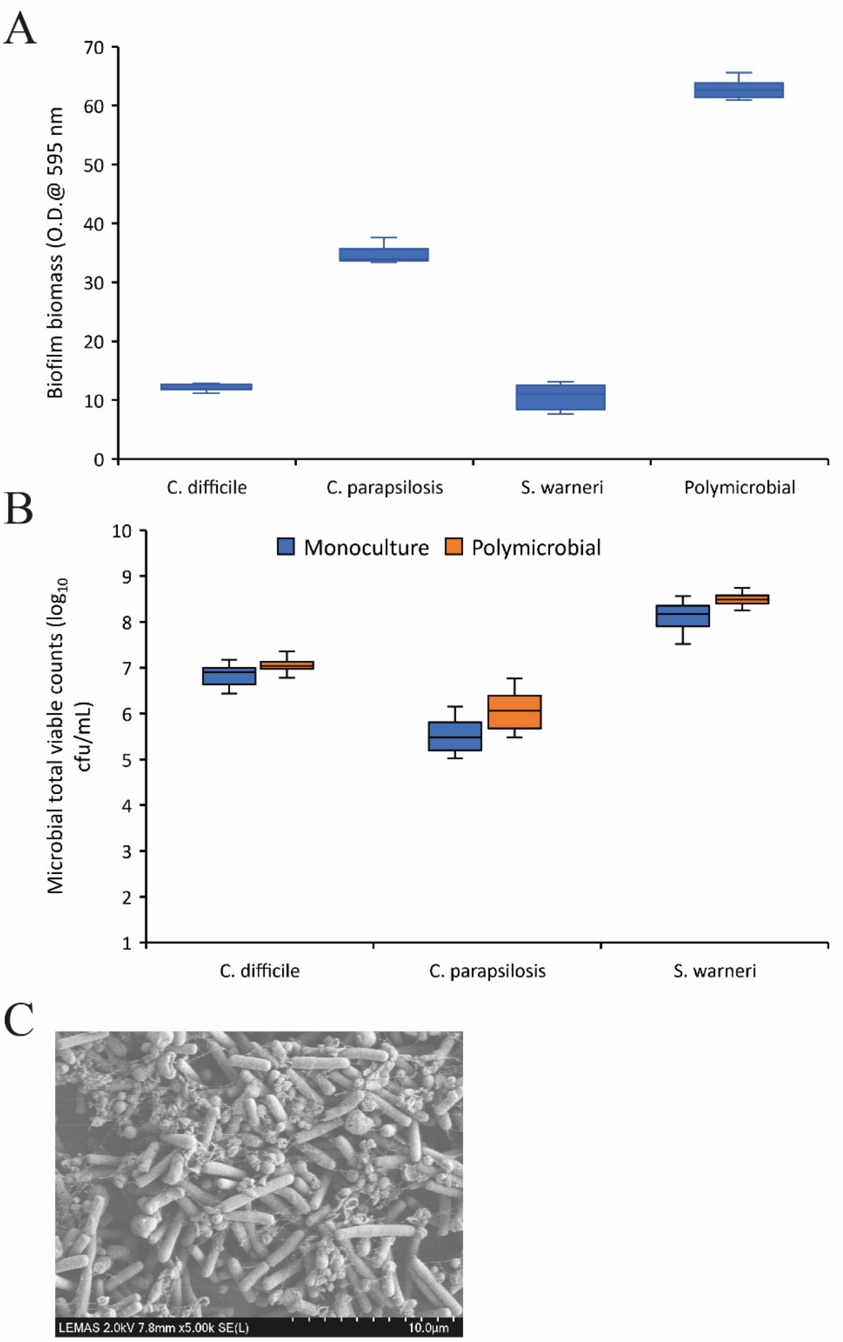

Supplementary figure 8

Effect of two different combinations of antagonistic and synergistic species on *C. difficile* biofilm formation. (**A**) Polymicrobial biofilms of *C. difficile*, *Candida albicans* (synergistic) and *Lactobacillus rhamnosus* (antagonistic), and (**B**) Polymicrobial biofilms of *C. difficile*, *Bacteroides thetaiotaomicron* (synergistic) and *Bifidobacterium longum* (antagonistic). Bacteria were enumerated from monoculture (blue) and polymicrobial (orange) biofilms using selective agars (Supplementary Table 1). Results shown are the total viable counts (cfu/mL) from three biological replicates and at least 12 technical replicates.

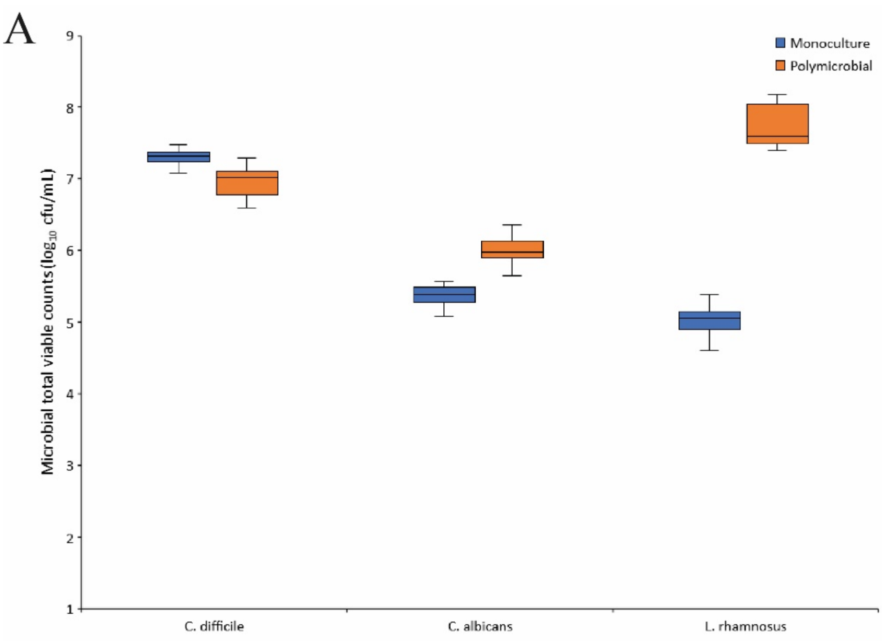

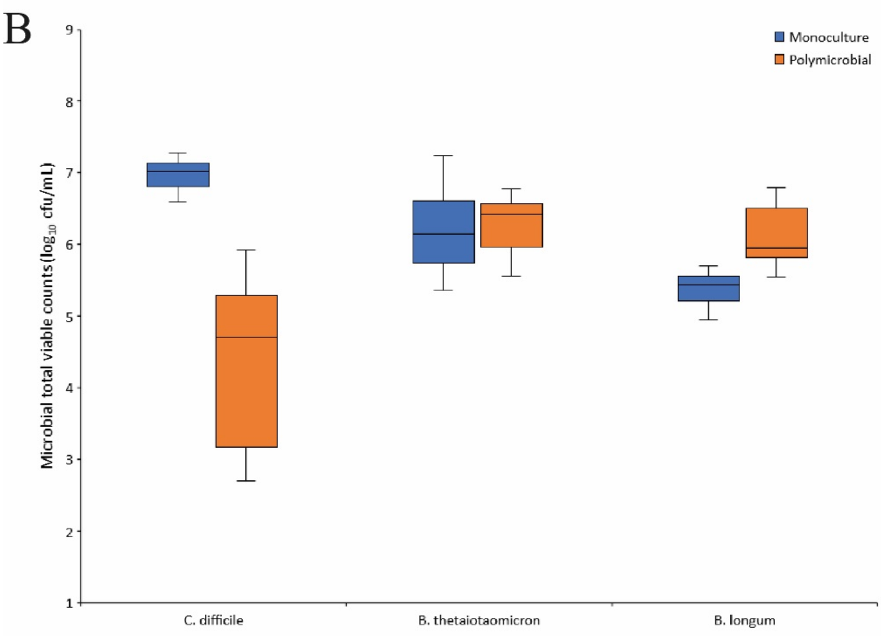

Supplementary table 1

Sessile microbial species identified by MALDI-TOF from the *in vitro* support structures from figure 2.

Acidaminococcus fermentans	*Enterococcus gallinarum*
Alistipes finegoldii	*Enterococcus mundtii*
Bacillus cereus	*Escherichia coli*
Bacillus mycoides	*Eubacterium limosum*
Bacillus thuringiensis	*Eubacterium plautii*
Bacteroides ovatus	*Hungatella hathewayi*
Bacteroides thetaiotaomicron	*Klebsiella oxytoca*
Bifidobacterium breve	*Klebsiella pneumoniae*
Bifidobacterium dentium	*Lactobacillus casei*
Bifidobacterium longum	*Lactobacillus delbrueckii*
Candida albicans	*Lactobacillus fermentum*
Candida krusei	*Lactobacillus gasseri*
Candida parapsilosis	*Lactobacillus harbinensis*
Citrobacter freundii	*Lactobacillus johnsonii*
Citrobacter koseri	*Lactobacillus kitasatonis*
Clostridioides difficile	*Lactobacillus parabuchneri*
Clostridium aminovalericum	*Lactobacillus paracasei*
Clostridium butyricum	*Lactobacillus reuteri*
Clostridium celerescrescens/sphenoides[a]	*Lactobacillus rhamnosus*
Clostridium clostridioforme	*Lysinibacillus boronitolerans*
Clostridium disporicum	*Lysinibacillus fusiformis*
Clostridium innocuum	*Lysinibacillus sphaericus*
Clostridium paraputrificum	*Lysinibacillus spp.*
Clostridium scindens	*Micrococcus luteus*
Clostridium sporogenes	*Morganella morganii*
Clostridium symbiosum	*Neisseria flavescens*
Clostridium tertium	*Parabacteroides distasonis*
Comamonas kerstersii	*Pseudomonas aeruginosa*
Eisenbergiella spp.	*Staphylococcus aureus*
Enterobacter asburiae	*Staphylococcus caprae*
Enterobacter cloacae	*Staphylococcus epidermidis*
Enterobacter kobei	*Staphylococcus haemolyticus*
Enterococcus avium	*Staphylococcus hominis*
Enterococcus casseliflavus	*Staphylococcus saprophyticus*
Enterococcus durans	*Staphylococcus warneri*
Enterococcus faecalis	*Staphylococcus gallolyticus*
Enterococcus faecium	*Tissierella spp.*
[a] *C. celerescrescens* and *C. sphenoides* share 98% genome homology and cannot be distinguished by MALDI-TOF	

Supplementary table 2

Agars used for direct enumeration and isolation of luminal and biofilm microbiota.

**Target organisms**	**Media and Supplements**	**Growth environment (@ 37 °C)**
C. difficile total viable counts	Brazier's CCEYL with 2% lysed horse blood, 5 mg/L lysozyme, 250 mg/L D-cycloserine, 8 mg/L cefoxitin, 2 mg/L moxifloxacin, 8 mg/L amphotericin B and 10 mg/L colisin	Anaerobic
C. difficile spores	1:1 ethanol (100%) shock for one hour followed by enumeration on Brazier's CCEYL with 2% lysed horse blood, 5 mg/L lysozyme, 250 mg/L D-cycloserine, 8 mg/L cefoxitin	Anaerobic
Total anaerobes and total *Clostridium* spp.	Pre-poured fastidious anaerobe agar (FAA) with 5 % horse blood	Anaerobic
Total *Clostridium* spores	1:1 ethanol (100%) shock for one hour followed by enumeration on pre-poured fastidious anaerobe agar (FAA) with 5 % horse blood	
Lactobacillus spp.	LAMVAB agar - 52.5 mg/L MRS broth and 20 mg/L agar technical with 0.5 g/L L-cysteine and 20 mg/L vancomycin	Anaerobic
Bifidobacterium spp.	Beerens agar - 42.5 mg/L Columbia agar and 5 mg/L agar technical with 5 mg/L glucose, 0.5 g/L L-cysteine and 5 ml propionic acid, adjusted to pH 5.	Anaerobic
Enterococcus spp.	Kanamycin aesculin azide agar with 10 mg/L nalidixic acid, 10 mg/L aztreonam, 20 mg/L kanamycin and 1 mg/L Lincomycin	Aerobic

Bacteriodes spp.	Bacteroides bile aesculin agar with 2% haemin and 0.002 % vitamin K1.	Anaerobic
Total facultative anaerobes	Pre-poured nutrient agar	Aerobic
Lactose-fermenting Enterobacteriaceae	Pre-poured MacConkey agar	Aerobic
Yeast and Mould spp.	Sabouraud Dextrose agar with 100 mg/L chloramphenicol	Aerobic (@ 22 °C)
