# Supplementary material for: Biofilms harbour Clostridioides difficile, serving as a reservoir for recurrent infection
Source: NPJ Biofilms Microbiomes. 2021 Feb 5;7:16. doi: 10.1038/s41522-021-00184-w (PMC7864922; doi:10.1038/s41522-021-00184-w)
Supplement: Supplementary file 2 — Reporting Summary [file 41522_2021_184_MOESM2_ESM.pdf]

nature research

Corresponding author(s): Dr Anthony Buckley

Reporting Summary

Nature Research wishes to improve the reproducibility of the work that we publish. This form provides structure for consistency and transparency in reporting. For further information on Nature Research policies, see Authors & Referees and the Editorial Policy Checklist.

Statistical parameters

When statistical analyses are reported, confirm that the following items are present in the relevant location (e.g. figure legend, table legend, main text, or Methods section).

n/a	Confirmed	
☐	☒	The exact sample size (*n*) for each experimental group/condition, given as a discrete number and unit of measurement
☐	☒	An indication of whether measurements were taken from distinct samples or whether the same sample was measured repeatedly
☐	☒	The statistical test(s) used AND whether they are one- or two-sided<br>*Only common tests should be described solely by name; describe more complex techniques in the Methods section.*
☒	☐	A description of all covariates tested
☐	☒	A description of any assumptions or corrections, such as tests of normality and adjustment for multiple comparisons
☐	☒	A full description of the statistics including central tendency (e.g. means) or other basic estimates (e.g. regression coefficient) AND variation (e.g. standard deviation) or associated estimates of uncertainty (e.g. confidence intervals)
☐	☒	For null hypothesis testing, the test statistic (e.g. *F*, *t*, *r*) with confidence intervals, effect sizes, degrees of freedom and *P* value noted<br>*Give P values as exact values whenever suitable.*
☒	☐	For Bayesian analysis, information on the choice of priors and Markov chain Monte Carlo settings
☒	☐	For hierarchical and complex designs, identification of the appropriate level for tests and full reporting of outcomes
☒	☐	Estimates of effect sizes (e.g. Cohen's *d*, Pearson's *r*), indicating how they were calculated
☐	☒	Clearly defined error bars<br>*State explicitly what error bars represent (e.g. SD, SE, CI)*

Our web collection on statistics for biologists may be useful.

Software and code

Policy information about availability of computer code

Data collection	*Provide a description of all commercial, open source and custom code used to collect the data in this study, specifying the version used OR state that no software was used.*
Data analysis	QIIME (version 1.9.1) was used for demultiplexing the 16S rRNA sequencing reads. DADA2 (version 1.4.0) was used to bin the amplicon sequences and to assign taxonomic function using the SILVA 16S rRNA database (SILVA nr v128).

For manuscripts utilizing custom algorithms or software that are central to the research but not yet described in published literature, software must be made available to editors/reviewers upon request. We strongly encourage code deposition in a community repository (e.g. GitHub). See the Nature Research guidelines for submitting code & software for further information.

Data

Policy information about availability of data

All manuscripts must include a data availability statement. This statement should provide the following information, where applicable:

- Accession codes, unique identifiers, or web links for publicly available datasets
- A list of figures that have associated raw data
- A description of any restrictions on data availability

The taxonomic data presented in Figure 2 is available from the Research Data Leeds Repository using the following link: https://doi.org/10.5518/784

Field-specific reporting

Please select the best fit for your research. If you are not sure, read the appropriate sections before making your selection.

☒ Life sciences ☐ Behavioural & social sciences ☐ Ecological, evolutionary & environmental sciences

For a reference copy of the document with all sections, see nature.com/authors/policies/ReportingSummary-flat.pdf

Life sciences study design

All studies must disclose on these points even when the disclosure is negative.

Sample size	No sample-size calculations were performed for the experiments in this paper. Where possible, we present data from three biological replicates; however, with gut model studies, this is not always possible due to the time and cost associated with each experiment.
Data exclusions	No data were excluded from these studies.
Replication	The results presented from gut model studies are from two biological repeats. The results from the in vitro biofilm assays were from at least three biological replicates.
Randomization	The results presented here are from laboratory analysis rather than a clinical trial, thus randomisation is not appropriate.
Blinding	The results presented here are from laboratory analysis rather than a clinical trial, thus blinding is not appropriate.

Reporting for specific materials, systems and methods

Materials & experimental systems

n/a	Involved in the study
☒	☐ Unique biological materials
☒	☐ Antibodies
☒	☐ Eukaryotic cell lines
☒	☐ Palaeontology
☒	☐ Animals and other organisms
☐	☒ Human research participants

Methods

n/a	Involved in the study
☒	☐ ChIP-seq
☒	☐ Flow cytometry
☒	☐ MRI-based neuroimaging

Human research participants

Policy information about studies involving human research participants

Population characteristics	Faecal donations from healthy volunteers were required to seed the gut models. Volunteers were recruited based on age (>59 years old) and no previous antibiotic history in the last 3 months.
Recruitment	Volunteers were recruited from the general public by reading a participant information sheet.
